# ID 66 - Etanercepte Referência e Biossimilar: análise da persistência em pacientes com artrite reumatoide no Sistema Único de Saúde (SUS)

**DOI:** 10.5327/2237-9622.2025.v34s1.66

**Published:** 2025-11-25

**Authors:** Adson José Moreira, Wallace Mateus Prata, Francisco de Assis Acurcio†, Augusto Afonso Guerra, Juliana Alvares-Teodoro

**Keywords:** terapia biológica, etanercepte, medicamentos biossimilares, persistência da medicação, artrite reumatoide

## Abstract

**Introdução:**

A artrite reumatoide (AR) é uma doença inflamatória crônica que pode levar à perda funcional e deterioração da qualidade de vida. O tratamento envolve medicamentos modificadores do curso da doença biológicos, eficazes no controle da doença, porém de custo elevado. Biossimilares foram desenvolvidos para reduzir custos e aumentar o acesso à terapia, mostrando-se seguros e eficazes. Estima-se que eles possam economizar até USD 54 bilhões em dez anos nos EUA. Apesar de a comprovação clínica da similaridade em eficácia e segurança, a avaliação do desempenho dos biossimilares em mundo real ainda é limitada. A persistência ao tratamento é um indicador de sucesso terapêutico e avaliá-la é essencial para encorajar o uso dos biossimilares, fornecendo dados que demonstrem sua equivalência com os medicamentos de referência. O objetivo é avaliar a persistência ao tratamento entre pacientes com AR iniciando terapia com o etanercepte de referência e biossimilar em um contexto de mundo real.

**Método:**

Coorte retrospectiva, de janeiro de 2020 até dezembro de 2021, com dados de pacientes diagnosticados com AR, de acordo com a Classificação Internacional de Doenças (CID), registrados na Sala Aberta de Inteligência em Saúde – Sabeis/DataSUS. Do total de 17.036 pacientes em uso de biológicos para o tratamento da AR, foram excluídos os seguintes pacientes: todos em uso de outros biológicos que não o etanercepte em monoterapia, os menores de 18 anos, os experimentados e aqueles com menos de dois registros de dispensação. A amostra foi dividida em dois grupos: pacientes usando o originador e pacientes usando o biossimilar. O teste de correlação de Pearson confirmou a homogeneidade entre os grupos em relação às variáveis de interesse. Foi avaliada a persistência global, do tempo zero até a interrupção ou troca de medicamento, por meio do método de Kaplan-Meier e as diferenças das curvas pelo log-rank; a persistência foi estratificada por sexo, faixa etária, região de residência, IMC, CID-10 e esquema terapêutico.

**Resultados:**

Dos 888 pacientes cadastrados na base Sabeis/DataSUS em uso de etanercepte em monoterapia como primeiro medicamento biológico, 543 estavam iniciando o originador e 345 iniciando o biossimilar. A amostra era composta na sua maioria por mulheres com idade entre 45 e 64 anos, predominantemente residentes na Região Sudeste. A análise de persistência não diferenciou os biossimilares dos biológicos originadores, e não foram observadas diferenças significativas entre os dois grupos. Na análise univariada, variáveis como sexo, idade, região de residência, IMC, CID-10 e esquema terapêutico não apresentaram significância estatística em relação ao risco de descontinuação do medicamento. No entanto, pacientes residentes nas Regiões Sul, Sudeste e Nordeste demonstraram um risco reduzido de descontinuação ao tratamento quando comparados aos residentes da Região Centro-Oeste.

**Conclusão:**

Este estudo de mundo real em âmbito nacional mostrou que o etanercepte biossimilar e o originador possuem efetividade e segurança equivalentes em pacientes com artrite reumatoide, confirmando os resultados observados em estudos realizados em outros países. Esses achados podem contribuir para a redução dos custos de tratamento no âmbito do Sistema Único de Saúde, dessa forma aumentando o acesso ao cuidado, promovendo equidade e melhorando a sustentabilidade do sistema de saúde.

